# The Pattern of Procalcitonin in Primary Total Hip and Knee Arthroplasty and its Implication in Periprosthetic Infection

**DOI:** 10.4021/jocmr2009.04.1236

**Published:** 2009-05-20

**Authors:** Syed Ali, Andrew Christie, Andrew Chapel

**Affiliations:** a SHO 3 Orthopaedics, Inverclyde Royal InfirmaryHospital, Greenock., PA16 0XN, Glsgow, U.K; b SHO 3 Orthopaedics, Western Infirmary, Glasgow, U.K; c Inverclyde Royal Infirmary Hospital, Greenock., PA16 0XN, Glasgow, U.K

## Abstract

**Background:**

The serum marker Procalcitonin (PCT) has been shown to be a sensitive indicator of bacterial infection, but very little is known of its behavior in periprosthetic infection. In this study, PCT was compared with standard tests used to aid the diagnosis of infection. As a baseline, its pattern in uncomplicated hip and knee arthroplasty was investigated.

**Methods:**

A prospective study of fifty-nine patients had bloods taken preoperatively, and on days 1, 3, 5, for PCT, C- reactive protein, erythrocyte sedimentation rate and white cell count.

**Results:**

Fifty patients (85%) had normal PCT values (< 0.5 ng/ml) and only 5 recorded a value > 1.0ng/ml. On day 5 only 1 patient had a value > 0.5ng/ml. The standard tests all showed sporadic elevations over the 3 days. PCT levels are not significantly elevated by the trauma of this surgery, as they are in other surgical procedures.

**Conclusions:**

PCT may be very useful in patients with suspected periprosthetic infection.

**Keywords:**

Infection; Periprosthetic; Procalcitonin; C-reactive protein; Arthroplasty

## Introduction

Two of the major complications of total knee and hip joint arthroplasty is peri-prosthetic infection and aseptic loosening. Many studies have investigated means of differentiating the two, mainly because the approach to revision arthroplasty and prognosis differs significantly between them. However, the diagnosis of peri-prosthetic infection still remains a difficult diagnosis [1].

Part of the explanation for this is the lack of reliability of the commonly used blood tests- C-reactive protein (CRP), erythrocyte sedimentation rate (ESR) and white cell count (WCC). A spectrum of inflammation naturally occurs in response to the tissue injury caused by surgery, commonly named the acute phase response. It has been shown that these blood parameters lack sensitivity and specificity in discriminating the inflammation of infection per se from that of the acute phase response [2-7].

Since the early 1990s there has been much interest addressing whether procalcitonin (PCT) is a more specific marker in detecting early bacterial infection in the perioperative period. Although they have often given conflicting results, it is largely believed that PCT is a more accurate indicator of infection [8-10]. PCT is a precursor of the hormone calcitonin, and is detectable in the serum of healthy individuals at a concentration of less than 0.5 ng/ml. Bacterial endotoxin has been shown to directly release PCT into the circulation [11, 12].

However, it is also elevated by the acute phase response [8], and this has resulted in much debate in the literature as to what cut-off value should represent infection [3, 13]. This has caused ambiguous conclusions in different studies regarding the superiority of PCT. This has been largely seen in abdominal and cardiothoracic surgery, as well as intensive care medicine. This is partly because these operations commonly cause a severe inflammatory response including the Systemic Inflammatory Response Syndrome-in non-infectious cases [8, 14-16].

Very few studies have looked at PCT in orthopaedics, and have only included small subgroups of patients in groups of patients undergoing different types of surgery [17]. Thus it appeared worthwhile to perform a study looking specifically at the characteristics of PCT in orthopaedic surgery. The aim of this study is therefore twofold: to illustrate the pattern of PCT in comparison to the routine inflammatory markers and, from this, to show if PCT could have a clinical role in periprosthetic infection.

## Materials and Methods

A prospective study over 6 months of fifty-nine patients undergoing either primary total hip or knee arthroplasty was performed. The mean age was 70.5 years, involving 19 men and 40 women. There were no exclusion criteria, and the study was sponsored by NHS Argyll and Clyde. Written informed consent was obtained. Prophylactic cefuroxime was given at induction of anesthesia followed by 2 post-operative doses.

Serum blood samples for PCT, CRP, ESR and WCC were taken pre-operatively and on days 1, 3 and 5 post-operatively. Blood pressure, pulse, respiratory rate and temperature were also recorded on these days. None of the patients showed any clinical sign of infection during their stay in hospital.

Hospital records were reviewed after their routine 6 week follow-up appointments. All the patients had been subsequently referred to the nurse lead arthroplasty clinic, indicating no clinical suspicion of infection.

The data was analysed by a statistician. PCT was measured using the Lumitest PCT-Q (Brahms Diagnostica, Germany).

## Results

The descriptive statistics of the measured variables are shown in Tables 1 and 2.

**Table 1:** Descriptive statistics of the average values for the parameters over the 3 post-operative days

Variable	Mean	St Dev	Minimum	Q1	Median	Q3	Maximum
AVE PCT	0.5096	1.117	0.3	0.3	0.3	0.3	10
AVE CRP	87.28	61.02	10	39	83	121	292
AVE ESR	51.36	31.37	4	25.5	48	77	125
AVE WCC	9.672	2.579	4.6	7.75	9.5	11.1	18.3

**Table 2:** Descriptive statistics for each parameter on all days

Variable	Mean	St Dev	Minimum	Q1	Median	Q3	Maximum
Day 0 PCT	0.3797	0.6119	0.3	0.3	0.3	0.3	5
Day 1 PCT	0.4051	0.3496	0.3	0.3	0.3	0.3	2
Day 3 PCT	0.798	1.877	0.3	0.3	0.3	0.3	10
Day 5 PCT	0.3254	0.1027	0.3	0.3	0.3	0.3	1
Day 0 CRP	11.78	9.64	2	10	10	10	62
Day 1 CRP	75.32	47.22	12	38	66	98	189
Day 3 CRP	129.02	62.44	10	91	120	162	292
Day 5 CRP	57.49	48.47	10	20	46	91	280
Day 0 ESR	20.76	17.33	4	7	14	26	77
Day 1 ESR	31.42	25.59	4	13	25	42	121
Day 3 ESR	62.56	30.48	4	41	59	88	125
Day 5 ESR	60.1	28.16	4	42	52	82	122
Day 0 WCC	7.731	2.329	3.4	6.1	7.8	9	17.5
Day 1 WCC	10.261	2.842	5	8.3	10	11.8	18.3
Day 3 WCC	9.922	2.229	5.7	7.9	9.8	10.9	18.3
Day 5 WCC	8.834	2.451	4.6	7	8.8	10.2	16.3

### PCT

**Figure 1:**
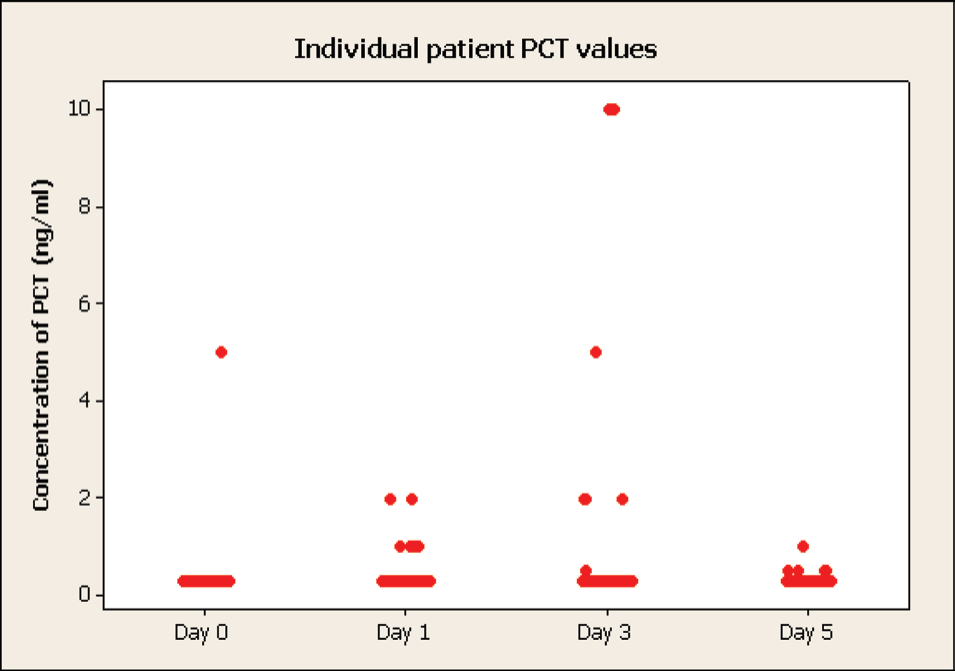
Concentrations of PCT.

Fifty patients (85%) had concentrations of PCT within the normal range (< 0.5 ng/ml) on all four days. In the remaining nine patients there was no significant day of occurrence of the maximum PCT level, and only five patients (8%) recorded a value > 1.0 ng/ml. Two patients had a value > 5 ng/ml. This was measured at 10 ng/ml on day 3. These patients had values of < 0.5 ng/ml on the other 3 days. One patient had a preoperative value > 0.5 ng/ml. Only 1 patient had a value > 0.5 ng/ml on day 5. The data is shown on Figure 1.

### CRP, ESR and WCC

Nine patients had a preoperative CRP value above the normal range (< 10 mg/l ). The day of occurrence of the maximum value was day 3 in 71% of patients, day 1 in 20% and day 5 in 9%. The maximum recorded value was 292 mg/l on day 3. This patient's CRP was < 10mg/l on day 5.

Thirteen patients had an ESR above a reference range of 30 mm/hr preoperatively. Days 3 and 5 had an equal distribution of patient's maximum values, (46% on each day).

**Figure 2:**
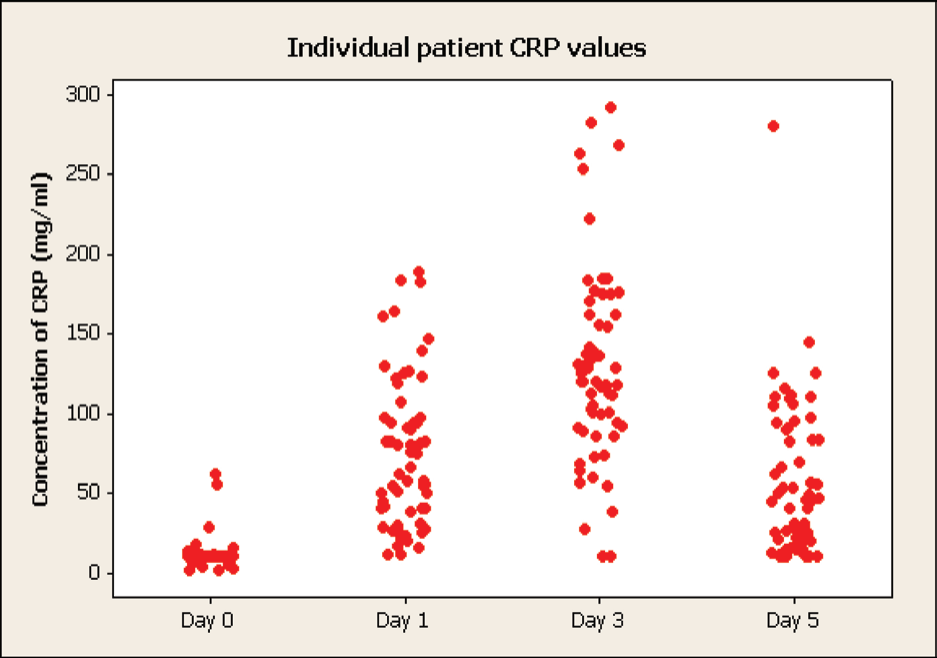
Changes of C-reactive protein.

**Figure 3:**
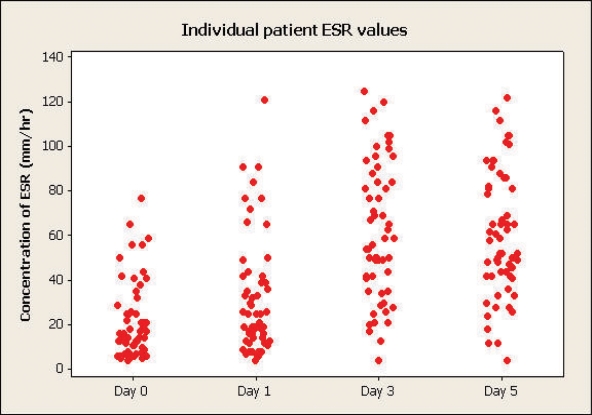
Changes of erythrocyte sedimentation rate (ESR).

**Figure 4:**
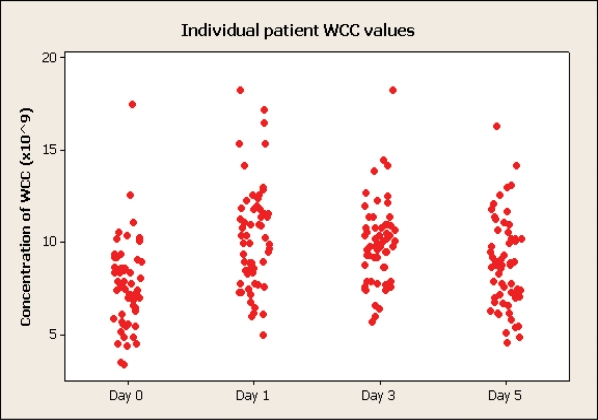
Changes of white cell count (WCC).

Two patients had a preoperative WCC >12000/mm^3^, and five patients (8%) had there maximum value on this day. Twenty nine (49%) had there maximum value on day 1, 34% on day 3 and 9% on day 5. This data is illustrated on Figures 2-4.

## Discussion

Procalcitonin is a polypeptide prohormone of calcitonin, mainly produced by the c-cells of the thyroid (8). It was initially described as a possible marker of infection in 1993 [18]. Since then there has been growing interest in the question of whether is a more specific marker of bacterial infection. Although it gas been shown to respond directly to bacterial endotoxin [11, 12], it is also stimulated by non-infective inflammation [8-10, 14, 15, 19, 20]. This has caused conflicting opinion regarding its superiority in this matter. Unsurprisingly, many papers have drawn comparisons with CRP. A meta-analysis of such papers [9] concluded that PCT was a more accurate marker of bacterial infection. However, many well designed studies have shown that PCT is no better than CRP [21-23].

There is further dispute in surgery because the severity of the natural inflammatory response (acute phase response) depends upon the type of operation. There is much debate as to what cut-off value of PCT differentiates infection from the acute phase response [3, 13]. Most studies have been in cardio-thoracic and abdominal surgery where cut-off values vary from 1- 10 ng/ml during the perioperative period [5, 9, 13, 15-17]. This is largely because these procedures commonly cause the systemic inflammatory response syndrome [8, 16, 24].

None of the patients in this study developed systemic inflammatory response syndrome (SIRS). The results convincingly show that hip and knee arthroplasty don't cause a significant rise in PCT. Measurements for diagnostic purposes are more helpful when the surgical trauma itself does not trigger considerable PCT release.

It is also proposed that PCT is better than CRP in the perioperative period because secretions begin within 4 hours and peak at 8 hours. CRP peaks only after 36 hours [9, 25]. The behavior of CRP in hip and knee arthroplasty is well documented- a pronounced rise at day 2 or 3, followed by a sharp decline [25, 26]. These results show this pattern, suggesting that this group is typical representation of other such studies. The results also indicate the limited use of ESR and WCC in this setting. Interestingly, nine patients (15%) had elevated CRP levels preoperatively. The only significance in their medical background was that three had previously undergone either hip or knee arthroplasty. The PCT was normal in these patients. Further studies have looked into other serum markers, one such paper analysing interleukin-6 in periprosthetic infection [2]. They had to exclude several patients because this test is elevated by chronic inflammatory diseases. It would appear from this study that PCT isn't affected in this way.

In summary, PCT may be a more reliable indicator of periprosthetic infection given the substantial proportion of patients with unexplained high CRP levels preoperatively. Also, because the surgery doesn't cause PCT to rise indicates that it could be used to monitor high risk patients in the immediate post- operative period. This is further supported by its rapid elevation in infection, and the fact that the CRP, ESR and WCC are still elevated during this period. This would further imply that PCT would be useful in monitoring patients after their revision surgery for periprosthetic infection when it is often difficult to know if the infection has been eradicated [4, 7]. A large multicentre study involving patients undergoing revision surgery for infection needs to be performed to validate these findings and assumptions.

## References

[1] Patel R, Osmon DR, Hanssen AD (2005). The diagnosis of prosthetic joint infection: current techniques and emerging technologies. Clin Orthop Relat Res.

[2] Di Cesare PE, Chang E, Preston CF, Liu CJ (2005). Serum interleukin-6 as a marker of periprosthetic infection following total hip and knee arthroplasty. J Bone Joint Surg Am.

[3] Mitaka C (2005). Clinical laboratory differentiation of infectious versus non-infectious systemic inflammatory response syndrome. Clin Chim Acta.

[4] Itasaka T, Kawai A, Sato T, Mitani S, Inoue H (2001). Diagnosis of infection after total hip arthroplasty. J Orthop Sci.

[5] Falcoz PE, Laluc F, Toubin MM, Puyraveau M, Clement F, Mercier M, Chocron S (2005). Usefulness of procalcitonin in the early detection of infection after thoracic surgery. Eur J Cardiothorac Surg.

[6] van Leeuwen MA, van Rijswijk MH (1994). Acute phase proteins in the monitoring of inflammatory disorders. Baillieres Clin Rheumatol.

[7] Rafiq M, Worthington T, Tebbs SE, Treacy RB, Dias R, Lambert PA, Elliott TS (2000). Serological detection of Gram-positive bacterial infection around prostheses. J Bone Joint Surg Br.

[8] Whicher J, Bienvenu J, Monneret G (2001). Procalcitonin as an acute phase marker. Ann Clin Biochem.

[9] Simon L, Gauvin F, Amre DK, Saint-Louis P, Lacroix J (2004). Serum procalcitonin and C-reactive protein levels as markers of bacterial infection: a systematic review and meta-analysis. Clin Infect Dis.

[10] Arkader R, Troster EJ, Lopes MR, Junior RR, Carcillo JA, Leone C, Okay TS (2006). Procalcitonin does discriminate between sepsis and systemic inflammatory response syndrome. Arch Dis Child.

[11] Dandona P, Nix D, Wilson MF, Aljada A, Love J, Assicot M, Bohuon C (1994). Procalcitonin increase after endotoxin injection in normal subjects. J Clin Endocrinol Metab.

[12] Reinhart K, Karzai W, Meisner M (2000). Procalcitonin as a marker of the systemic inflammatory response to infection. Intensive Care Med.

[13] Di Filippo A, Lombardi A, Ognibene A, Messeri G, Tonelli F (2002). Procalcitonin as an early marker of postoperative infectious complications. Minerva Chir.

[14] Rothenburger M, Markewitz A, Lenz T, Kaulbach HG, Marohl K, Kuhlmann WD, Weinhold C (1999). Detection of acute phase response and infection. The role of procalcitonin and C-reactive protein. Clin Chem Lab Med.

[15] Baykut D, Schulte-Herbruggen J, Krian A (2000). The value of procalcitonin as an infection marker in cardiac surgery. Eur J Med Res.

[16] Mimoz O, Benoist JF, Edouard AR, Assicot M, Bohuon C, Samii K (1998). Procalcitonin and C-reactive protein during the early posttraumatic systemic inflammatory response syndrome. Intensive Care Med.

[17] Meisner M, Tschaikowsky K, Hutzler A, Schick C, Schuttler J (1998). Postoperative plasma concentrations of procalcitonin after different types of surgery. Intensive Care Med.

[18] Assicot M, Gendrel D, Carsin H, Raymond J, Guilbaud J, Bohuon C (1993). High serum procalcitonin concentrations in patients with sepsis and infection. Lancet.

[19] Stucker F, Herrmann F, Graf JD, Michel JP, Krause KH, Gavazzi G (2005). Procalcitonin and infection in elderly patients. J Am Geriatr Soc.

[20] Selberg O, Hecker H, Martin M, Klos A, Bautsch W, Kohl J (2000). Discrimination of sepsis and systemic inflammatory response syndrome by determination of circulating plasma concentrations of procalcitonin, protein complement 3a, and interleukin-6. Crit Care Med.

[21] Ugarte H, Silva E, Mercan D, De Mendonca A, Vincent JL (1999). Procalcitonin used as a marker of infection in the intensive care unit. Crit Care Med.

[22] Suprin E, Camus C, Gacouin A, Le Tulzo Y, Lavoue S, Feuillu A, Thomas R (2000). Procalcitonin: a valuable indicator of infection in a medical ICU?. Intensive Care Med.

[23] Hambach L, Eder M, Dammann E, Schrauder A, Sykora KW, Dieterich C, Kirschner P (2002). Diagnostic value of procalcitonin serum levels in comparison with C-reactive protein in allogeneic stem cell transplantation. Haematologica.

[24] Pittet D, Rangel-Frausto S, Li N, Tarara D, Costigan M, Rempe L, Jebson P (1995). Systemic inflammatory response syndrome, sepsis, severe sepsis and septic shock: incidence, morbidities and outcomes in surgical ICU patients. Intensive Care Med.

[25] Foglar C, Lindsey RW (1998). C-reactive protein in orthopedics. Orthopedics.

[26] Niskanen RO, Korkala O, Pammo H (1996). Serum C-reactive protein levels after total hip and knee arthroplasty. J Bone Joint Surg Br.

